# Preoperative FLAIR images for identifying glioblastoma boundaries

**DOI:** 10.1186/s12880-025-01839-2

**Published:** 2025-07-30

**Authors:** Bayan Shukir, Laszlo Szivos, Pal Barzo, David Kis

**Affiliations:** 1 Neurosurgery Department, Medicine Faculty, University of Szeged, Szeged, Hungary; 2 Basic Science Department, College of Pharmacy, University of Duhok, Kurdistan Region, Iraq

**Keywords:** Glioblastoma, FLAIR, Contrast-enhanced T1MRI, Tumor progression

## Abstract

**Background:**

Glioblastoma is the most aggressive and rapidly growing type of central nervous system tumor. Despite advancements in imaging, no objective measurement for predicting the true extent of glioblastoma has been established. Compared with contrast-enhanced magnetic resonance imaging (MRI), fluid-attenuated inversion recovery (FLAIR) imaging is believed to be more sensitive for detecting infiltrated tumor cells. This study investigates the sensitivity and specificity of preoperative FLAIR imaging to predict glioblastoma true boundaries.

**Methods:**

Our study was retrospectively registered enrolling 20 high-grade glioma patients whose data from 16 patients were analyzed. For each patient, the primary tumor mask was identified on the preoperative FLAIR image covering the whole hyperintense region. Tumor cells infiltration mask was defined on follow-up MRI representing where the tumor recurred. According to automated anatomical labeling 3 (AAL3) and Johns Hopkins University, international consortium of brain mapping, diffusion tensor imaging-white matter-81 labels (JHU ICBM-DTI-81) standard atlases, standard brain was divided into cortical and subcortical regions. Sensitivity and specificity were determined counting the number of brain areas overlapped by the preoperative FLAIR tumor mask and the recurrence tumor mask.

**Results:**

The overall sensitivity and specificity was 82.6%, and 84.7%, respectively. Individually, hyperintensity on FLAIR images demonstrated high sensitivity but low specificity in some cases, while in others, the opposite pattern was observed. To validate the reliability of our method, predictive values were defined. The group average positive predictive value and negative predictive value were 50% and 96%, respectively.

**Conclusion:**

Although FLAIR imaging demonstrates potential in delineating the extent of glioblastoma, its predictive value remains unclear, emphasizing the need for supplementary methodologies to enhance tumor delineation and improve clinical outcomes.

**Clinical trial number:**

Not applicable.

## Background

Glioblastoma is the most malignant central nervous system tumor, with a median survival 12–15 months [1–3]. Maximal safe resection followed by chemoradiotherapy is the standard treatment for glioblastoma [4]. Despite advances in imaging, no objective method to define glioblastoma true boundaries has been established yet.

Standard magnetic resonance imaging (MRI) protocols, including contrast-enhanced T1, T2, diffusion weighted imaging (DWI), and fluid-attenuated inversion recovery (FLAIR), are commonly utilized for tumor visualization and intraoperative surgical-guiding to assist with tumor resection [5–7]. Contrast-enhanced T1-weighted (CE-T1) MRI is often referred as the most effective imaging modality to visualize glioblastoma [8–10]. Glioblastoma typically presents as a heterogeneous mass with a necrotic center and irregular contrast enhancement [11]. Components beyond contrast enhancing margins regarded as noncontrast-enhanced tumors, could also evolve to a fast growing contrast enhanced tumor threatening survival. These microscopic infiltrated tumor cells are usually difficult to be detected on CE-T1 [8, 10, 12]. In the last decade, FLAIR imaging has been included in glioblastoma assessment, suggesting that postcontrast enhancement alone is not sufficient in evaluating radiographic disease due to the infiltrative glioblastomas’ nature [9]. FLAIR changes on imaging have been suggested to have an increased sensitivity in detecting microscopic tumor cells and noncontrast enhancing lesions [13]. Hyperintensity on FLAIR imaging often extends beyond the contrast-enhancing portion of a glioblastoma tumor, and recently an increased interest is focused into the tumor microenvironment composition within areas of FLAIR change [14–16]. It is demonstrated that the hyperintense region observed on FLAIR imaging does not primarily indicate tumor presence, but rather than reflects a composite signal of both microscopic tumor cells and edema [17]. However, findings of other studies propose that FLAIR signal abnormality in glioblastoma contains infiltrative tumor cells and survival rate correlates to the amount of this abnormality srrounding glioblastoma [18], and more than 90% of tumor recurrences will occur within this hyperintense region [19]. In addition, the high-signal intensity of fluid within the resection cavity observed on follow-up FLAIR images considered as a hallmark of tumor progression. This property of FLAIR play a key role in the tumor progression and tumor recurrence [20–22].

Most glioblastoma studies have focused on the removing the contrast-enhancing tumor part by introducing various approaches in attempt to improve patient survival and outcomes [23–25]. Regardless the strong evidence provided by these studies, evaluating the removal of noncontrast-enhancing tumor on patient outcomes was neglected.

Several studies have sought to correlate patient outcomes and maximal safe resection of both contrast-enhancing tumor and noncontrast-enhancing FLAIR; however, the results varied among the groups. Results have shown that increasing the extent of resection of the FLAIR abnormality beyond the contrast-enhancing tumor associated with best outcomes [26–28]. On the other hand, Altieri and team results showed that the extent of FLAIR signal abnormality does not correlate with survival [29]. The variability of these results could be resulted in how the pre-lesional noncontrast-enhancing tumor was defined in the literature. Despite the effectiveness of FLAIR as imaging tool to localize glioblastomas, planning radiation treatment, and post-treatment monitoring, its prognostic value remains unclear [30].

The majority of studies focus on invistigating the abnormality volume observed on FLAIR images as a potential indicator of prolonged survival. To the best of our knowledge, no previous study has assessed to explore the sensitivity and specificity of preoperative FLAIR imaging for delineating glioblastoma true boundaries. In this study, we introduce a new insights into this critical aspect by evaluating the reliability of sensitivity and specificity of preoperative FLAIR imaging for accurately identifying glioblastoma true extent.

## Patients and methods

### Study design

The methodology followed in this study for defining masks and image analysis was adapted from Kis et al. [31] with moderate modifications to incorporate sensitivity, specificity, and predictive values calculations for preoperative FLAIR imaging.

The segmentation techniques and registration algorithms implemented in this study were followed as described previously by Kis and team (2022), adjusting them to evaluate regional overlap between preoperative FLAIR and follow-up tumor recurrence masks.

### Study population

Our study included 20 high-grade glioma patients who were treated between 2010 and 2021. The inclusion criteria were as follows: (1) histologically confirmed high-grade glioma, (2) age > 18 years, (3) subtotal or total tumor resection, (4) availability of preoperative FLAIR imaging, and follow-up MRI. Exclusion criteria were for those patients who underwent partial resection or incomplete follow-up data.

Patients’ clinical data are presented in Table 1. [see Additional file 1].

**Table 1:** Patients clinical data

Patients	Age (years) and Sex	Histology	Localization	Side	Time of Diagnosis	Date of Recurrence	Date of Last Follow-up/Death	PFS (months)	OS (months)
1	26 Male	Glioblastoma	Frontalis	Right	2010.03	2012.08	2014.01	28	46
2	54 Female	Glioblastoma	Frontalis	Left	2010.04	2013.02	2013.03	34	35
3	39 Male	Glioblastoma	Parito-occipitalis	Right	2010.07	2012.05	2014.06	22	47
4	47 Male	Glioblastoma	Frontalis	Left	2010.09	2011.01	2012.05	4	20
5	34 Male	Glioblastoma	Frontalis	Left	2010.12	2011.05	2011.10	6	11
6	53 Male	Glioblastoma	Tempro-paritalis	Left	2011.02	2011.06	2012.02	4	12
7	51 Female	Oligodendroglioma-G3	Paritalis	Left	2011.02	2016.03	2017.02	61	72
8	67 Male	Glioblastoma	Temporalis	Left	2012.02	2012.07	2013.02	5	12
9	29 Male	Oligodendroglioma-G3	Fronto-paritalis	left	2012.05	2016.04	2017.09	47	64
10	67 Female	Oligodendroglioma-G3	Frontalis	Right	2012.06	2015.07	2015.07	37	37
11	68 Male	Glioblastoma	Temporalis	Left	2013.01	2014.02	2014.09	13	20
12	46 Male	Glioblastoma	Frontalis	Right	2019.11	2020.03	2021.02	4	15
13	77 Female	Glioblastoma	Tempro-paritalis	Left	2019.10	2020.06	2020.08	8	10
14	46 Female	Glioblastoma	Paritalis	Left	2021.01	2022.01	2022.02	1	2
15	55 Male	Glioblastoma	Tempro-parito-occipitalis	Right	2021.02	2021.04	2021.06	2	4
16	53 Male	Glioblastoma	Frontalis	Left	2021.10	2021.12	2022.02	2	3

PSF: progression free survival, OS: overall survival [31]

## Data acquisition and processing

MRI scans were performed via 3 Tesla MRI GE SIGNA Excite scanners (GE Healthcare, Chicago, IL, USA). Preoperative FLAIR and postoperative contrast-enhanced T1 MRI sequences were obtained with the following parameters:

FLAIR: 3D CUBE; TR/TE, 6,000/140 ms; FOV, 25 × 25 cm; slice thickness 1 mm; and matrix, 256 × 256.

Postoperative follow-up: contrast-enhanced high-resolution 3D IR-FSPGR; TR/TE/TI, 10.3/4.2/450 ms; flip angle, 15º; ASSET, 2; FOV, 25 × 25 cm; matrix, 256 × 256; and slice thickness 1 mm.

According to a previously published method, raw MRI data were processed and analyzed using the Functional MRI of the Brain Software Library (FSL, version 5.0.7; Oxford Centre for Functional MRI of the Brain (FMRIB), United Kingdom)^1^ [32]. DICOM files were converted to NIFTI format via MRICron software [33].

## Defining masks

In this study, two types of masks were defined: tumor masks (primary tumor and tumor recurrence masks) and, cortical and subcortical white matter masks. 

Primary tumor masks were defined on preoperative FLAIR image by delineating the whole hyperintense region reflecting both edema and infiltrative tumor cells.

The first follow-up MRI scans were performed postoperatively within 48–72 hours. According to the standard oncological follow-up protocol described by [9], subsequent follow-up scans were conducted at regular intervals (typically every 2–3 months) allowing a comparative assessments of tumor progression or recurrence.

**Fig. 1:**
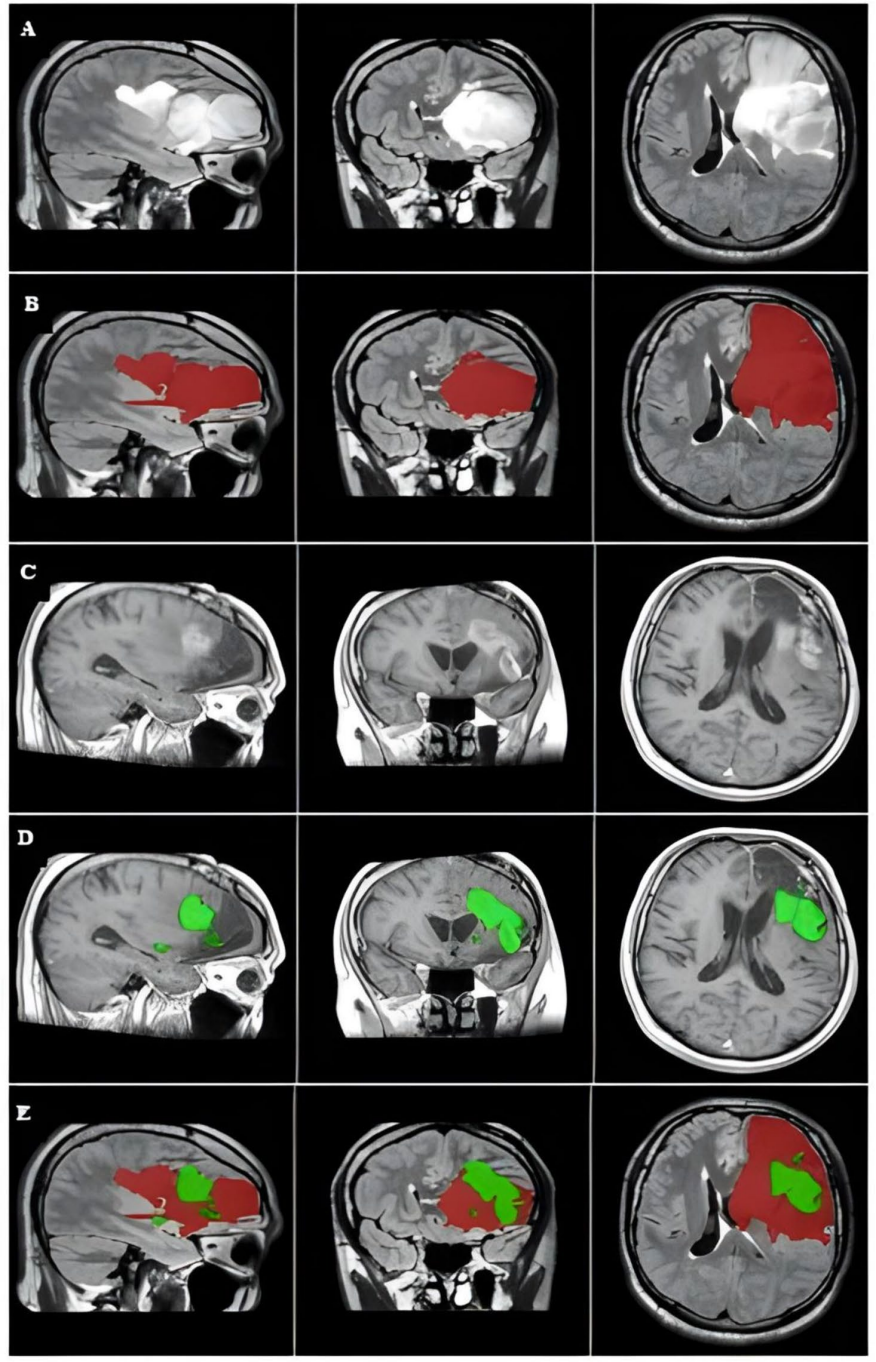
The primary tumor, tumor recurrence, and overlap between the two masks of a representative case. The images were captured in the MNI152 1-mm space. (**A**) The primary tumor is visualized on preoperative FLAIR image of the left parietooccipital area. (**B**) Preoperative FLAIR mask covering the hyperintense regions (red). (**C**) The size and location of tumor location around the resected cavity. (**D**) The tumor recurrence mask covers the postcontrast-enhanced area where the tumor recurred (green). (**E**) Axial plane showing the overlap between the primary tumor (red) and tumor recurrence (green)

**Fig. 2:**
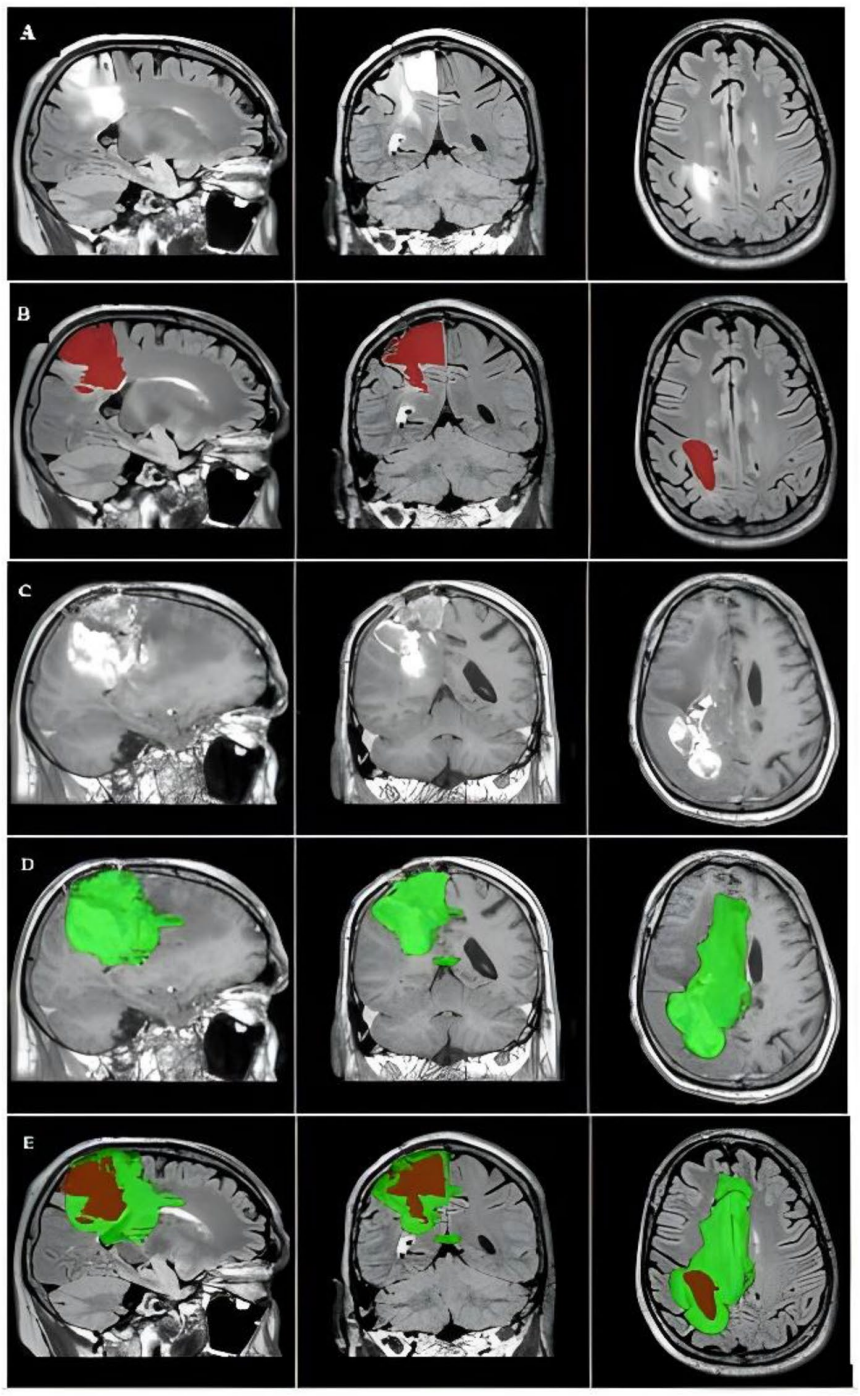
The primary tumor, tumor recurrence, and overlap between the two masks of a representative case. The images were captured in the MNI152 1-mm space. (**A**) The primary tumor is visualized on preoperative FLAIR image of the right frontal area. (**B**) Primary tumor mask covers the abnormal region on the preoperative FLAIR image (red). (**C**) Follow-up CE-T1 shows tumor progression, with the (**D**) contrast-enhanced tumor recurrence mask on CE-T1 where the tumor recurred (green). (**E**) Axial plane shows the overlap between the primary tumor (red) and tumor recurrence (green)

Follow-up CE-T1 MRI scans were used to identify tumor recurrence masks, revealing areas of new or increasing contrast enhancement. These areas are often correspond to regions of active tumor infiltration, in which typically interpreted as indicative of tumor prognosis [34, 35]. (Figs. 1 A-D, 2 A-D)

**Fig. 3:**
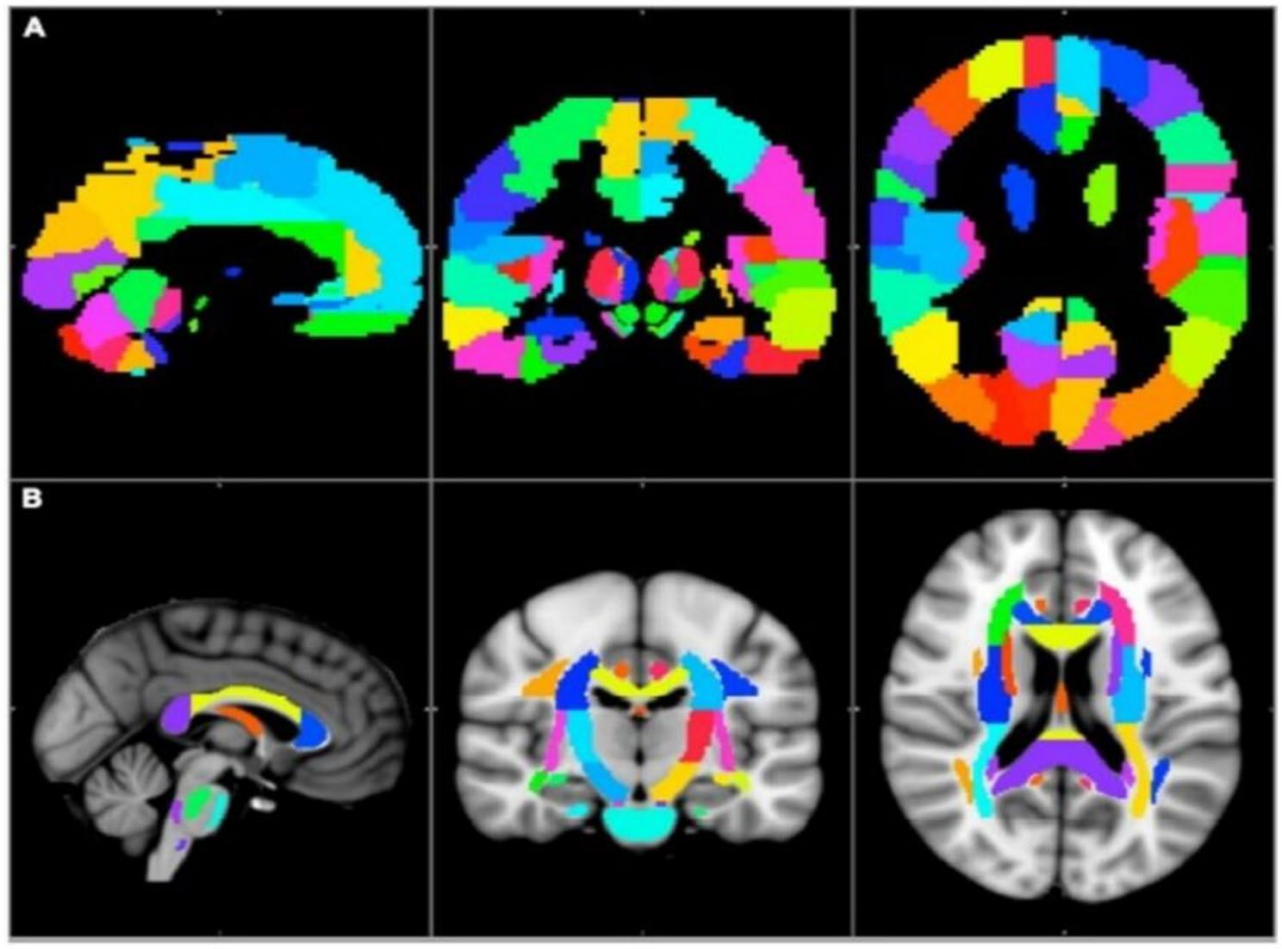
Brain regions in the MNI152 1-mm space. The (**A**) AAL3 cortical and (**B**) JHU–ICBM–DTI–81 white matter subcortical labels [31]

To identify brain regions infiltrated by the primary tumor and those involved in tumor recurrence, cortical and subcortical white matter masks were utilized. According to the standard maps, the standard brain was divided into distinct subcortical and cortical subregions as follows:

The standard Johns Hopkins University, international consortium of brain mapping, diffusion tensor imaging (JHU ICBM-DTI-81 white matter) map was used to segment the white matter into 54 subregions in right and left hemispheres each, and 6 midline structures [36]. The standard automated anatomical labeling 3 (AAL3) map was utilized to segment the cortex into 84 subregions on either side [37]. (Fig. 3). Both of these maps are in the standard MNI152 1 mm space (Montreal Neurological Institute, MNI152 1 mm brain).

## Image analysis

For analysis in the standard space, affine registration (12 degree of freedom, cost function: correlation ratio, interpolation: trilinear) of each patient’s preoperative FLAIR and follow-up FSPGR-T1 to the standard T1 images (Montreal Neurological Institute, MNI152 1 mm brain) was done. Image registration was performed with FMRIB’s linear registration tool (FLIRT) [38].

For each patient, the standard cortical and subcortical brain regions were projected onto the standard preoperative FLAIR tumor mask and the tumor recurrence mask it was then assessed how many of these regions affected by the two tumor masks. Subsequently, calculating sensitivity, specificity, positive predictive value (PPV), and negative predictive value (NPV) [39], (Figs. 1 E and 2 E), as follows:

$${\rm{Sensitivity = A/}}\left( {{\rm{A + C}}} \right)$$

$${\rm{Specificity = D/}}\left( {{\rm{D + B}}} \right)$$

True positives (A): number of brain regions covered by both the primary tumor and tumor recurrence masks.

False positives (B): number of brain regions covered only by the primary tumor mask.

False negatives (C): number of brain regions covered only by the tumor recurrence mask.

True negatives (D): number of brain regions not covered by the primary tumor mask or tumor recurrence mask.

Predictive values were defined as the proportion of true positives and true negatives using the following formulas:

$${\rm{PPV = A/}}\left( {{\rm{A + B}}} \right)$$

$${\rm{NPV = D/}}\left( {{\rm{D + C}}} \right)$$

## Results

This retrospective study included 20 patients whom diagnosed with high-grade glioma (13 glioblastomas, 3 oligodendeogliomas grade 3), classified according to the WHO 2021 criteria. Our final analysis included 16 patients (5 females, 11 males; median age, 52 years). The remaining 4 patients have been excluded according to the previously mentioned exclusion criteria. These 4 patients’ data have not been assessed neither individually nor included in the group level calculations.

Tumors were predominantly located in the left frontal lobe. The median survival time was 17.5 months (range, 2–72 months).

The two most common neurological deficit was speech disturbancies (25%) and hemiparesis (35%). Preoperatively, a total of 40% of the patients did not have severe neurological symptoms. Preoperatively, the average KPS performance was 73% and 80% at 2 months after the operation. (Medtronic Inc StealthStation iNav or S8) navigating tool used to plan individual minimal invasive craniotomy in all cases. All patients had either total (70%) or subtotal (30%) resection. The age, and female to- male ratio, progression-free and overall survival periods corresponded to the literature [40, 41].

Overall, the sensitivity was 82.6%, specificity was 84.7%, PPV was 50%, and NPV was 95.8%. Two patients showed high sensitivity and low specificity, and vice versa (Figs. 1 and 2), illustrating FLAIR’s diagnostic performance.

## Discussion

FLAIR imaging has become integral to glioblastoma assessment, particularly for visualizing infiltrating tumor cells beyond contrast-enhanced regions. According to the literatures, peritumoral edema typically appears as FLAIR hyperintensity surrounding the main tumor mass. In glioblastoma, this hyperintensity often reflects tumor cell infiltration into the surrounding brain parenchyma. However, differentiating where infiltrating tumor cells boundaries ends within edema is very challenging on FLAIR imaging.

Advanced imaging techniques, such as MR perfusion, diffusion imaging, spectroscopy, positron emission tomography, and DTI, are widely utilized in preoperative planning to enhance tumor visualization and maximizing safety of tumor resection. In particular, the DTI probabilistic tractography-based method introduced by Kis et al. [31] demonstrated promising results, with high sensitivity and specificity in predicting glioblastoma boundaries preoperatively. Following this concept, our study utilized FLAIR images as a base method to assess the extent of glioblastoma prior to surgery.

In our study, a standardized image registration and region-based comparison approach implemented, rather than relying on a direct volumetric overlap comparisons between pre- and postoperative tumor volumes. In this study, we came up with a method that focused on identifying brain regions overlapped by both the preoperative tumor and follow-up tumor recurrence masks.

To achieve our research objectives, several steps were undertaken. Firstly, preoperative and follow-up tumor masks were manually delineated on the preoperative FLAIR and follow-up CE-T1 sequences, respectively. Secondly, each patient’s preoperative FLAIR tumor mask and follow-up tumor mask were registered to the standard MNI152 1 mm space. This step was performed utlizing FMRIB’s linear registration tool (FLIRT). Finally, the standard brain regions also in the standard MNI152 1 mm space were overlapped onto the standard preoperative FLAIR tumor and the tumor recurrence masks, it was then defined how many of them covered by both tumor masks. Subsequently, calculating the sensitivity and specificity.

Following the abovementioned method, we avoided the anatomical variability across patients, enabling a region-based analysis.

In this framework, only the number of overlapped brain regions covered by the preoperative and postoperative tumor masks was considered critical. Tumor volume, precise tumor location, and scanning time were not prioritized; for the two main reasons. First, temporal changes in tumor volume are common; tumor progression observed on follow-up MRI often differs significantly from the initial tumor volume defined on preoperative FLAIR imaging. Consequently, a direct overlap comparison between preoperative FLAIR-defined tumor volume and tumor recurrence volume frequently results in temporal mismatches and unreliable spatial alignment between anatomical brain regions. See Figs. 1E and 2E. Even with the advanced sophisticated registration algorithms, identical alignment is not possible [42]. Second, the timing of the follow-up scan is critical. Sensitivity and specificity are directly affected by when the follow-up MRI is performed; a delay in scanning may lead to inconsistent results in detecting tumor progression or recurrence. Considering these modality-specific limitations and the variability in sensitivity and specificity, a direct volumetric comparison between preoperative FLAIR and follow-up images was deemed unreliable for our study.

Following the abovementioned steps, our results revealed no significant difference between overall sensitivity and specificity at 82.6% and 84.7% respectively.

Although our method demonstrated high sensitivity and specificity, it did not provide a clear estimate of the true false-negative-to-true false-positive ratio within FLAIR imaging. Specifically, the method does not assess the reliability of hyperintensity on FLAIR imaging in accurately distinguishing between tumoral and non-tumoral areas, such as edema. In glioblastoma surgery, the ratio of these factors are crucial because overestimating the extent of tumor resection may cause severe neurological impairments, whereas missing tumoral tissues directly affects survival. Therefore, additional statistical methods are needed to validate our theory.

PPVs and NPVs are defined to reflect the proportions of true positive and true negative results [39]. The group level PPV of 50.2% and NPV of 95.8% indicated that although FLAIR imaging was highly reliable in ruling out most of the nontumoral tissues (i.e., high NPV), it had a moderate likelihood of accurately confirming the presence of infiltrating tumor cells (i.e., low PPV). This discrepancy was likely attributable to a relatively high false-positive rate, which may have led to an overestimation of the tumor extent.

Before relying solely on preoperative FLAIR imaging, the two representative cases in which contradicting results were obtained should be considered.

In the first representative case (Patient 3, Fig. 1), the high sensitivity implied that most of the brain regions covered by the primary tumor mask were true cancerous tissues. This case supports the previously proposed reports that FLAIR imaging is very sensitive for assessing tumor cell proliferation. However, according to the literatures the hyperintense region on FLAIR may overestimate tumor boundaries by reflecting other factors, such as, edema, potentially leading to inaccuracies in diagnostic or treatment planning.

In contrast, the second representative case (Patient 4, Fig. 2) exhibited considerably higher specificity than sensitivity. Compared with the original tumor mass, the significantly larger area covered by the tumor recurrence mask indicated that the majority of the brain regions left undetected by the preoperative FLAIR tumor mask were, in fact, infiltrated by cancer cells that had spread into the surrounding tissue, ultimately led to rapid tumor progression, resulting in poor overall survival.

This study highlighted both the strengths and the limitations of preoperative FLAIR imaging in predicting glioblastoma proliferation. The two representative cases demonstrated that while hyperintensity in FLAIR images can visualize abnormal regions, it cannot accurately or reliably detect microscopic infiltrated tumor cells. With, it lacks the accuracy or reliability needed to detect microscopic infiltrated tumor cells. Furthermore, our results showed that although FLAIR-based predictive values can reliably rule out tumor-free tissues, they may fail to detect microscopic tumor cell infiltration, which can later propagate and form new contrast-enhancing lesions near the resected cavity, potentially impacting patient survival.

The inconsistency of our results could be mainly accounted for by several limitations, including manual delineation of tumor masks introduces potential variability, registration of different modalities into the standard image leading to anatomical misalignment, and the small sample size. Future studies incorporating a larger sample size and addressing the aforementioned limitations may lead to more reliable calculations.

In conclusion, integrating FLAIR imaging into routine glioblastoma assessment may enhance surgical precision and patient outcomes by limiting both overtreatment and tumor recurrence risks. However, our findings suggest that FLAIR-based predictive values, as assessed by sensitivity and specificity, remain limited. Therefore, we recommend incorporating FLAIR with advanced multimodal imaging to improve the accuracy of tumor boundary prediction in clinical practice.

## Data Availability

The datasets used and analyzed during the current study were adapted from the previously published study: [Predicting the True Extent of Glioblastoma Based on Probabilistic Tractography, Frontiers in Neuroscience, 2022, 10.3389/fnins.2022.886465.eCollection2022. As indicated in the original publication, the data are included in the article and supplementary materials. Further inquiries can be directed to the corresponding author of the original study]. ^(477)^

## Footnotes

1 http://www.fmri.oxac.uk/fsl

## Abbreviations

T1: T1 weighted imaging
T2: T2 weighted imaging
FLAIR: Fluid Attenuated Inversion Recovery
MRI: Magnetic resonance imaging
FMRIB: Functional magnetic resonance imaging of the brain
FSPGR: Fast spoiled gradient echo
TR: Time of repetition
TE: Time of echo
ASSET: Array coil spatial sensitivity encoding
FOV: Field of view
DICOM: Digital imaging and communications in medicine
DWI: Diffusion weighted images
DTI: Diffusion tensor images
SWI: Susceptibility weighted images
FSL: FMRIB software library
NIFTI: Neuroimaging informatics technology initiative
MNI152 1mm: Montreal neurological institute 152 1 millimeter
AAL3: Automated anatomical labeling atlas 3
JHU-ICBM-DTI: Johns Hopkins University, international consortium of brain mapping, diffusion tensor imaging
DOF: Degree of freedom
PPV: Positive predictive value
NPV: Negative predictive value
FLIRT: FMRIB’s linear registration algorithm
WHO: World Health Organization
