# Correction: Preoperative FLAIR images for identifying glioblastoma boundaries

**DOI:** 10.1186/s12880-025-01880-1

**Published:** 2025-08-26

**Authors:** Bayan Shukir, Laszlo Szivos, Pal Barzo, David Kis

**Affiliations:** 1 Neurosurgery Department, Medicine Faculty, University of Szeged, Szeged, Hungary; 2 Basic Science Department, College of Pharmacy, University of Duhok, Kurdistan Region, Iraq

**Correction to: Shukir et al. BMC Medical Imaging (2025) 25:302**

10.1186/s12880-025-01839-2

Following the publication of the original article, the authors reported captions for Fig 1 and 2 were mistakenly processed as part of the text.

The correct captions are as follows:

**Fig. 1:**
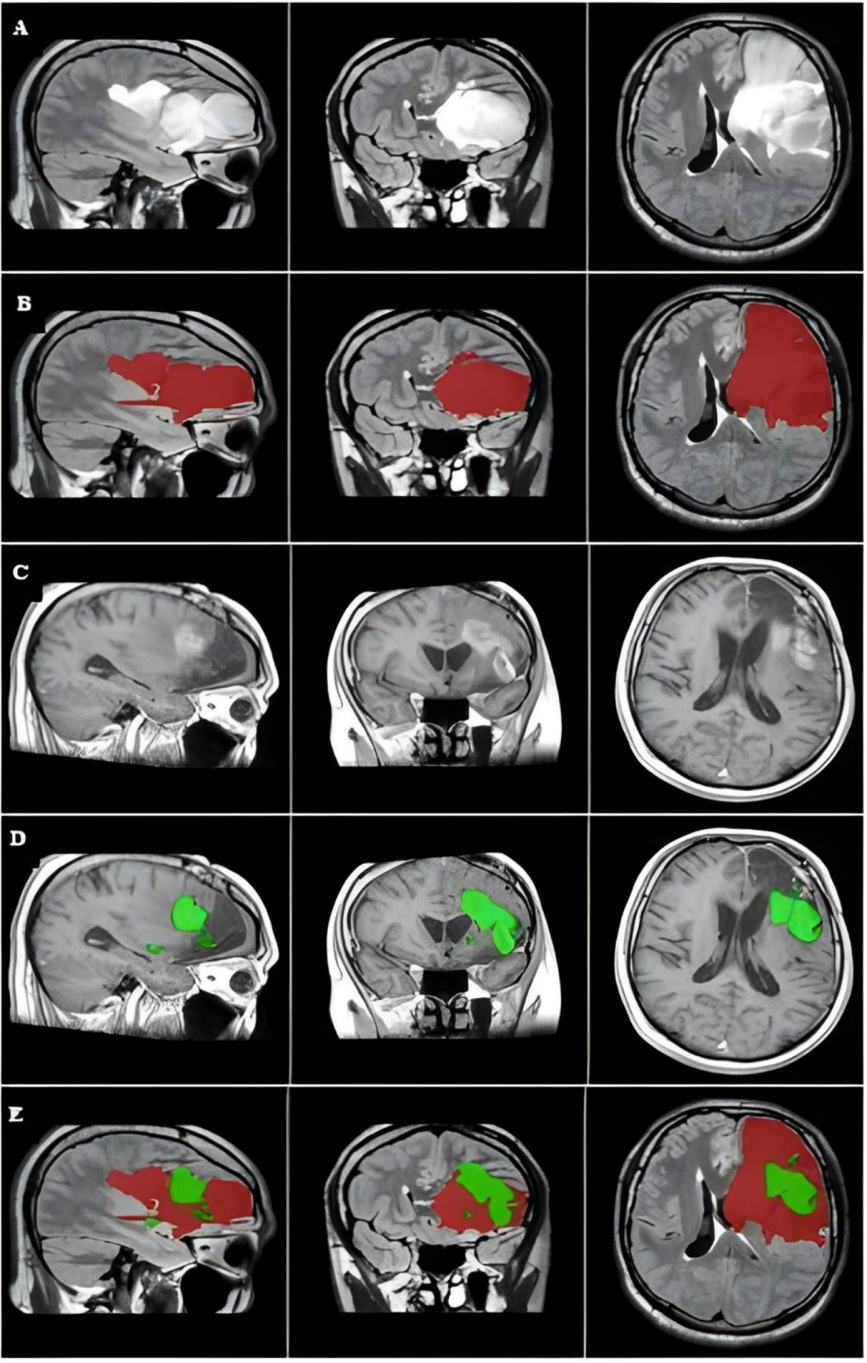
The primary tumor, tumor recurrence, and overlap between the two masks of a repesentative case. The images were captured in the MNI152 1-mm space. (**A**) The primary tumor is visualized on preoperative FLAIR image of the left parietooccipital area. (**B**) Preoperative FLAIR mask covering the hyperintense regions (red). (**C**) The size and location of tumor location around the resected cavity. (**D**) The tumor recurrence mask covers the postcontrast-enhanced area where the tumor recurred (green). (**E**) Axial plane showing the overlap between the primary tumor (red) and tumor recurrence (green)

**Fig. 2:**
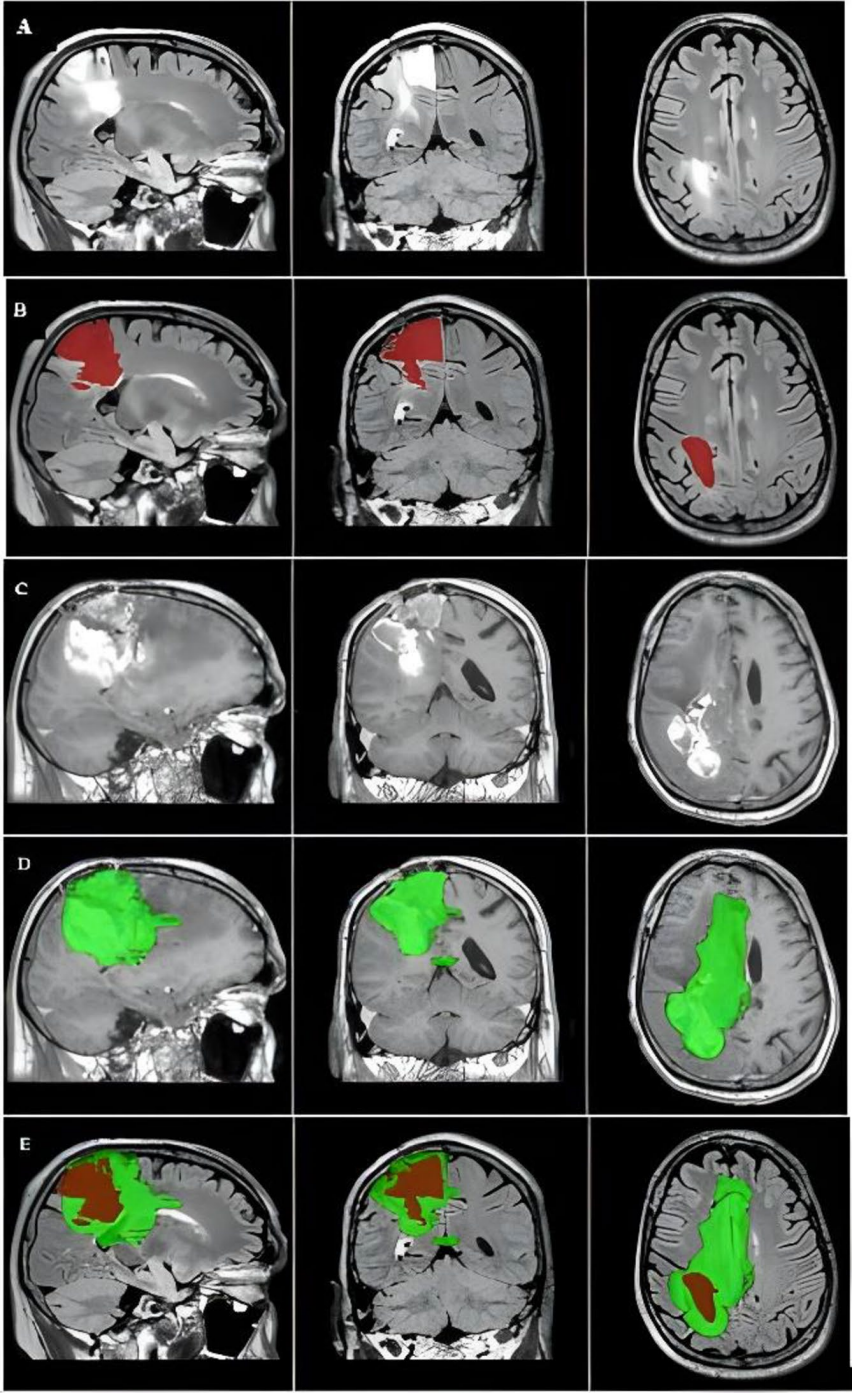
The primary tumor, tumor recurrence, and overlap between the two masks of a resresntative case. The images were captured in the MNI152 1-mm space. (**A**) The primary tumor is visualized on preoperative FLAIR image of the right frontal area. (**B**) Primary tumor mask covers the abnormal region on the preoperative FLAIR image (red). (**C**) Follow-up CE-T1 shows tumor progression, with the (**D**) contrast-enhanced tumor recurrence mask on CE-T1 where the tumor recurred (green). (**E**) Axial plane shows the overlap between the primary tumor (red) and tumor recurrence (green)

The original article has been updated accordingly.

